# Isolation, Identification, and Biological Activities of a New Chlorin e6 Derivative

**DOI:** 10.3390/ijms25137114

**Published:** 2024-06-28

**Authors:** Rameshwar Prasad Pandit, Til Bahadur Thapa Magar, Rajeev Shrestha, Junmo Lim, Pallavi Gurung, Yong-Wan Kim

**Affiliations:** 1 Dongsung Cancer Center, Dongsung Biopharmaceutical, Daegu 41061, Republic of Korea; prp08@ds-pharm.co.kr (R.P.P.); ijm15@ds-pharm.co.kr (J.L.); gp20@ds-pharm.co.kr (P.G.); 2 Center for Translational Science, Florida International University, 11350 SW Village Pkway, Port St. Lucie, FL 34987, USA; laphale@outlook.com; 3 Center for Food Animal Health, Department of Animal Sciences, The Ohio State University, Wooster, OH 44691, USA; shrestha.144@osu.edu

**Keywords:** chlorin e6, rhodin g_7_ 7^1^-ethyl ester, photosensitizer, photodynamic therapy, cancer cell lines, cytotoxicity

## Abstract

Chlorin e6 is a well-known photosensitizer used in photodynamic diagnosis and therapy. A method for identifying and purifying a novel process-related impurity during the synthesis of chlorin e6 has been developed. Its structure was elucidated using NMR and HRMS. This new impurity is formed from chlorophyll *b* rather than chlorophyll *a*, which is the source of chlorin e6. The intermediates formed during chlorin e6 synthesis were monitored using HPLC-mass spectrometry. This new impurity was identified as rhodin g_7_ 7^1^-ethyl ester, the structure of which remains unknown to date. The cytotoxic effects of this novel compound in both dark and light conditions were studied against five cancer cell lines (HT29, MIA-PaCa-2, PANC-1, AsPC-1, and B16F10) and a normal cell line (RAW264.7) and compared to those of chlorin e6. Upon irradiation using a laser at 0.5 J/cm^2^, rhodin g_7_ 7^1^-ethyl ester demonstrated higher cytotoxicity (2-fold) compared to chlorin e6 in the majority of the cancer cell lines. Furthermore, this new compound exhibited higher dark cytotoxicity compared to chlorin e6. Studies on singlet oxygen generation, the accumulation in highly vascular liver tissue, and the production of reactive oxygen species in MIA-PaCa-2 cancer cells via rhodin g_7_ 7^1^-ethyl ester correspond to its higher cytotoxicity as a newly developed photosensitizer. Therefore, rhodin g_7_ 7^1^-ethyl ester could be employed as an alternative or complementary agent to chlorin e6 in the photodynamic therapy for treating cancer cells.

## 1. Introduction

Chlorin e6 and its derivatives have received special attention due to their high photosensitizing ability [1] for use in photodynamic therapy (PDT) [2,3,4]. Chlorins treat the pathological tissue via the combined effect of excited photosensitizers (PSs) and molecular oxygen dissolved in the tissues [5]. Excited PSs then convert oxygen into highly cytotoxic reactive oxygen species (ROS) and singlet oxygen as well when exposed to light irradiation. As a result, ROS causes cell death and tissue destruction [6]. The cell type, PS type or concentration, intracellular localization, light dose, and oxygen partial pressure are the important factors that affect the cell death [7]. Chlorin e6 excited by using a 660–670 nm laser penetrates much deeper into human tissue than traditional first-generation PSs like Photofrin [8]. In addition, low dark toxicity, higher phototoxic potential, and selective tumor killing ability via immediate accumulation in the target tissue are notable features of chlorin e6 [9,10]. Therefore, chlorins have been used immensely in the diagnosis and treatment of various cancers such as pancreatic [11,12], lung [13], and bladder [14] and in microbial infections [15]. Despite the substantial advancements in PDT usage as a cancer therapy, it has certain limitations such as the buildup of photobleaching of photosensitizers, limited delivery of light doses, and ineffectiveness in tumor hypoxia [16].

Regarding the importance of chlorin e6, several synthetic methods have been reported [17,18,19] from chlorophyll *a*, pheophytin *a*, and pheophorbide *a*. In some instances, the degradation of chlorin e6 caused by heat, light, and oxidizing agents has been considered its synthetic method [20].

In order to develop an active pharmaceutical ingredient for drug formulation, an understanding of the impurities and degradation products in chlorin e6 is of paramount concern regarding the quality and safety of pharmaceuticals. Since the processing, manufacturing, and storage conditions of chlorin e6 may lead to degradation products which ultimately reduce the long shelf life of chlorin e6, the identification and effects of impurities in biological tissues have to be studied. Although there is a report on the isolation and identification of the impurities and degradation products in chlorin e6, studies on the process-related impurities are scarce [20,21].

In this work, we have developed a novel process-related impurity during the synthesis of chlorin e6. The identification, purification, and characterization method of it has also been discussed. The study aimed to discover whether several features, such as singlet oxygen generation, intracellular ROS formation, biodistribution, and cellular absorption distinguish chlorin e6 from its process-related product, rhodin g_7_ 7^1^-ethyl ester. Further, an assessment of the in vitro cancer potential of rhodin g_7_ 7^1^-ethyl ester in contrast to its parent chemical Ce6 was performed.

## 2. Results

The analysis of the process-related or degradations of the product was conducted considering the potential carry-over or conversion to other substances during the synthetic process steps. These evaluations are specifically important for drug substances which are obtained after several synthetic steps. These studies, therefore, support the justification of the quality and reliability of the active pharmaceutical ingredient. Here in this paper, we discuss the various aspects of isolation, purification, characterization, and evaluating a process-related impurity as an alternative to chlorin e6 for PDT based on the tested biological activities.

### 2.1. Isolation and Purification of Impurities

**Figure 1:**
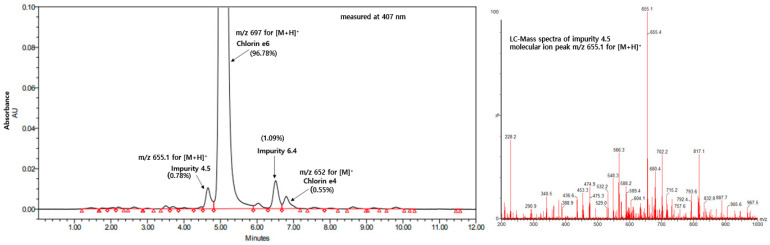
A typical HPLC chromatogram of chlorin e6 containing impurity 4.5 measured at 407 nm (**left**). Mass spectrum of impurity 4.5 (**right**).

Chlorin e6 was synthesized via the procedure mentioned in our earlier report [22,23] and we compared the data with that of commercially available chlorin e6 (obtained from Frontier) to establish its initial identification (Figure S1). Impurity 4.5 is a process-related impurity derived from chlorophyll *b* available in *Spirulina platensis* and undergoes modification during the process of chlorin e6 synthesis. The high-performance liquid chromatography of a typical batch of chlorin e6 with impurity 4.5 run on an analytical 150 mm × 4.6 mm reversed-phase column (Capcell Pak C18 UG120) packed with 5 µm particles is shown in Figure 1. In this figure, impurity 4.5 is named after the retention time in minutes, and the separation and purification of chlorin e6 and its degraded product (chlorin e4) were already performed [20]. Chlorin e4 is characterized as a degradation product of chlorin e6 and accumulates during the basic hydrolysis of the exocyclic ring. The mass spectrum of chlorin e4 showed a molecular ion with *m*/*z* 552 due to the decarboxylation of the least stable 15-acetic acid chain at C15 from the parent chlorin e6. The new impurity 4.5 appears to be 0.78% available in chlorin e6. During analysis, gradient elution was carried out using mobile phase A (0.1% TFA in water) and B (acetonitrile). Since impurity 4.5 was seen in a low content in the batch samples, we isolated impurity 4.5 from our stock sample stored in the dark, and it had a high impurity percentage due to multiple fractionation in a Biotage reversed-phase column. Reversed-phase column chromatography was performed on MPLC using a gradient solvent system. The desired compound was isolated at 36% of acetonitrile in 0.04% TFA in water. The yield of this impurity was found to be 0.007% (14 mg) from 200 g of *Spiriluna platensis*.

### 2.2. Identification and Structure Elucidiation of Impurity 4.5

**Figure 2:**
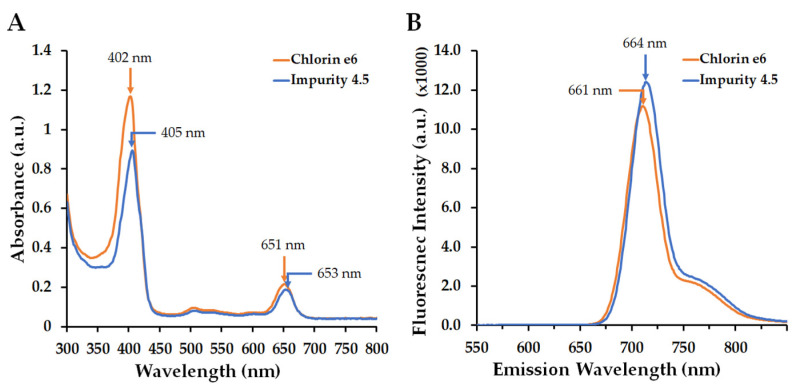
(**A**) UV–visible spectrum of impurity 4.5 and chlorin e6 and (**B**) emission spectrum of impurity 4.5 and chlorin e6. The test solutions were prepared in normal saline at a concentration of 20 ppm.

Qualitative analysis of impurity 4.5 was carried out at a 20 ppm concentration, and the absorbance was recorded in the range of wavelengths 300–800 nm and compared with that of chlorin e6 as shown in Figure 2A. The study of UV–visible spectral data indicates that impurity 4.5 has a similar absorbance band pattern to that of chlorin e6, showing the highest absorption peak at 402–406 (Soret band) and at 651–653 nm (Figure 2A). This has shown λ_max_ 653 of impurity 4.5 to redshift, thereby ensuring the presence of an additional conjugated π-system to the tetrapyrolic chlorin e6. Further, when both impurity 4.5 and chlorin e6 at 20 ppm were excited at λ 405 nm, the fluorescence emission spectra exhibited a fluorescence peak intensity at 664 nm for impurity 4.5 (Figure 2B).

A typical HPLC-MS analysis of chlorin e6 sample containing impurity 4.5 was analyzed as shown in Figure 1. The mass fragmentation of chorine e6 and the related structure are well known [21] and in most cases, are accompanied by decarboxylation. In a similar way, an intense peak for the molecular ion [M+H]^+^ of impurity 4.5 coupled with HPLC was found to be at 655.1, and the fragment [M-2CO_2_]^+^ at 566.3 was seen in the MS-ESI spectra due to the release of two carbon dioxide molecules at C13 and C15 [21] (Figure 1, right).

**Figure 3:**
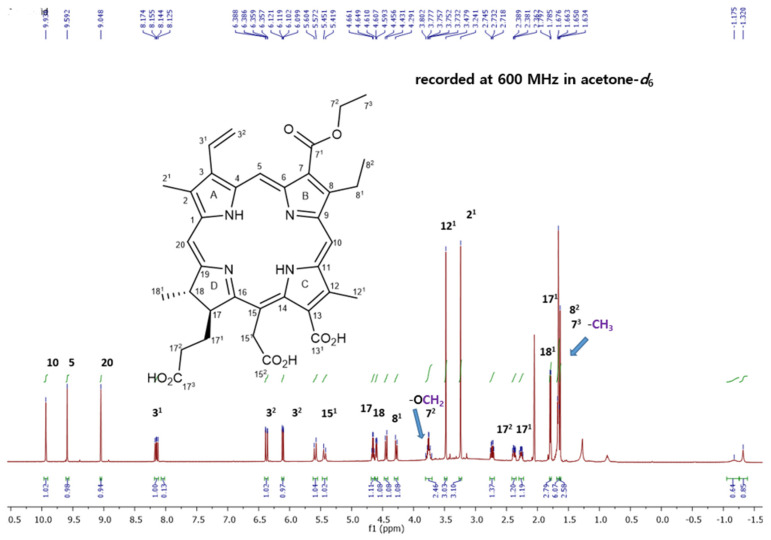
^1^H NMR spectrum of impurity 4.5.

**Figure 4:**
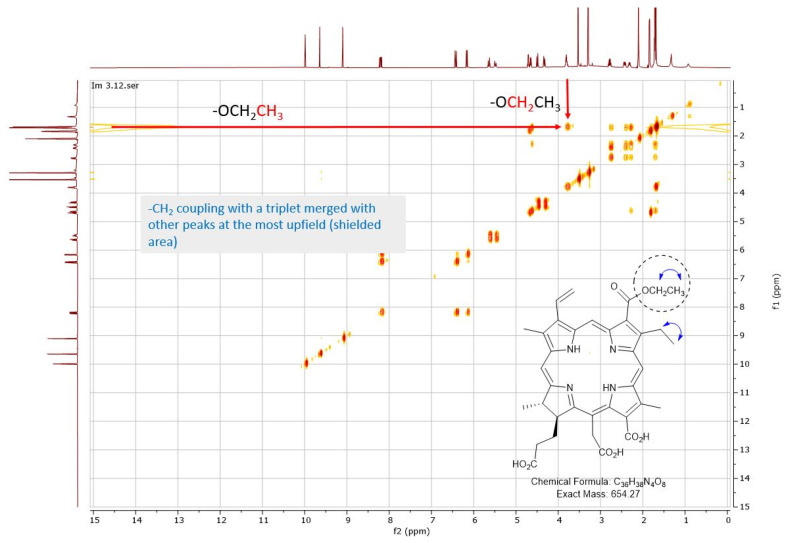
^1^H-^1^H cosy spectrum of impurity 4.5.

The structure of impurity 4.5 was identified via the analysis of its NMR and by comparison with the ^1^H NMR of chlorin e6 (Figure 3). The NMR of chlorin e6 has already been reported [22,23,24]. In the NMR of impurity 4.5, it was noted that one singlet peak corresponding to a methyl in chlorin e6 has been lost and appears as a multiplet corresponding to –OCH_2_ at 3.80–3.70 ppm in impurity 4.5. A triplet corresponding to an incoming ethoxy group appears to be merged at the very upfield. Further, the correlation of -OCH_2_ (3.7 ppm) with the adjacent -CH_3_ is seen at the most upfield, and the peak (for –CH_3_) merged with another triplet and a multiplet in the area around 1.6 ppm as shown in the ^1^H-^1^H cosy spectrum (Figure 4). The carbon NMR of impurity 4.5 taken at 125 MHz in acetone-*d*_6_ also supplements the structural characterization (Figure S2). Overall, an ethyl ester (-CO_2_Et) group is seen to be incorporated in the place of methyl of chlorin e6 at C7.

**Figure 5:**
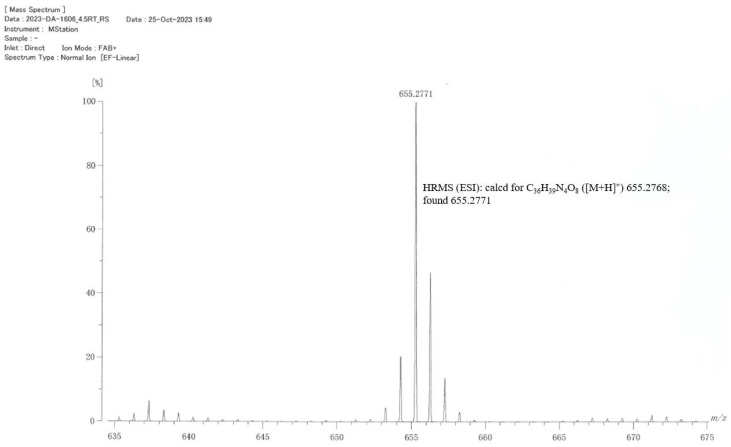
High-resolution mass spectrum of impurity 4.5.

While the calculated exact mass of the molecular ion [M+H]^+^ for impurity 4.5 was *m*/*z* 655.2768, the observed molecular ion peak for [M+H]^+^ at *m*/*z* 655.2771 via high-resolution mass spectrometry (HRMS) confirms the structure of impurity 4.5 as rhodin g_7_ 7^1^-ethyl ester (Figure 5).

### 2.3. The Scheme of the Formation of Rhodin g_7_ 7^1^-ethyl Ester

The known method of preparation of chlorin e6 involves the extraction of chlorophyll *a* in ethanol using the powder of *Spirulina platensis* as the first step. In doing so, it is not only chlorophyll *a* but also chlorophyll *b* and others (chlorophyll *a*’, pheophytin *a*, pheophytin *a*’, and carotenoids) that come into the extract, and thus, chlorophyll *b* turns out to be the ultimate source of rhodin g_7_ 7^1^-ethyl ester.

**Scheme 1:**
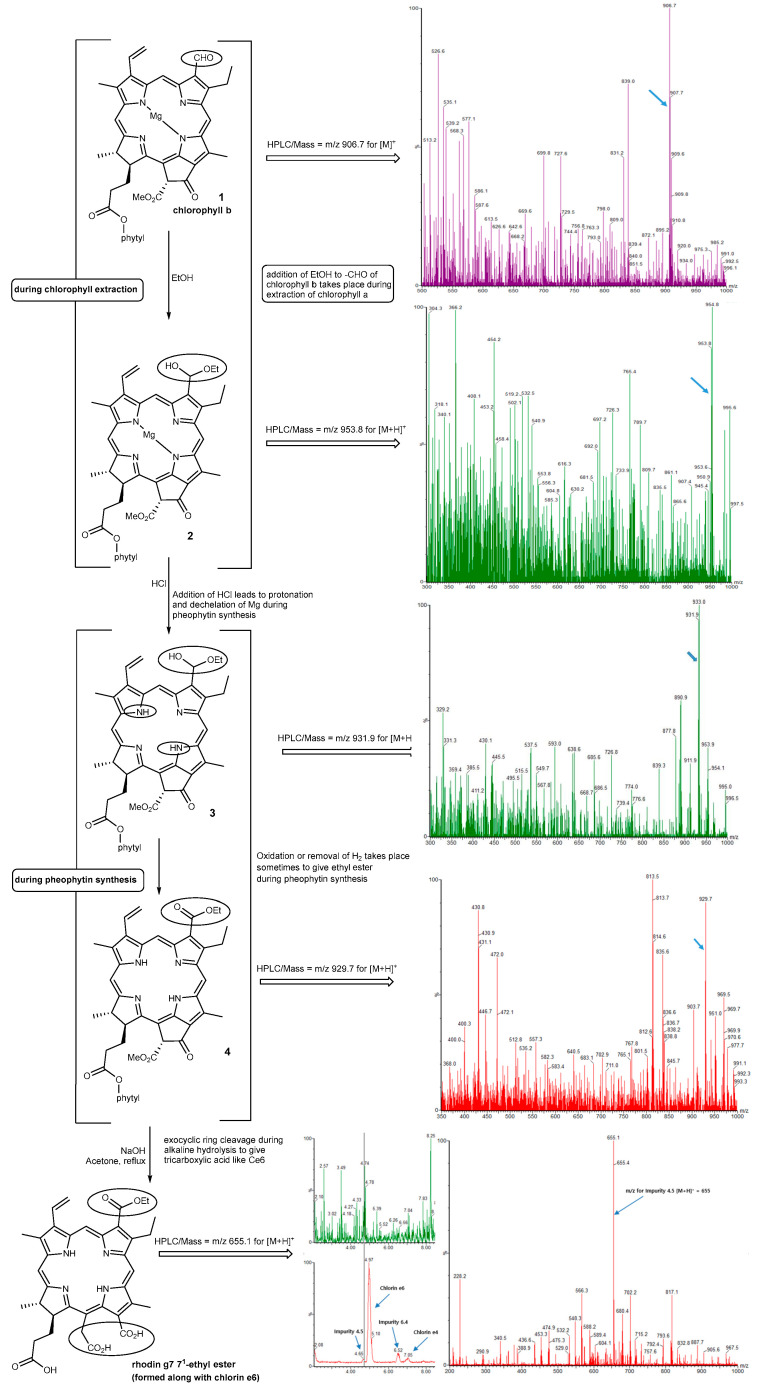
Proposed scheme outlining the mechanism for the formation of rhodin g_7_ 7^1^-ethyl ester. ESI-mass spectra of chlorophyll *b* and its derivatives were obtained throughout the synthesis of chlorin e6.

In our case, rhodin g_7_ 7^1^-ethyl ester is seen to be formed via the direct aldol reaction at the carbonyl (-CHO) of chlorophyll *b* which may be available in *Spirulina platensis* (Scheme 1). Since chlorophyll *b* (**1**) has an aldehyde functional group, it reacts with ethanol during liquid extraction to give (**2**). The additional product undergoes dechelation on acid treatment to give **3** which is formed during pheophytin synthesis. In the pheophytin synthesis, sometimes dehydrogenation of alcohol (**3**) takes place to give an ethyl ester (**4**). Product **4** undergoes hydrolysis of the exocyclic ring under basic treatment giving tricarboxylic acids such as rhodin g_7_ 7^1^-ethyl ester. This reaction is accompanied by the conversion of pheophytin a into chlorin e6.

Further, we propose a conceptual scheme for the industrial preparation of rhodin g_7_ 7^1^-ethyl ester (see Supplementary Material Figure S3).

### 2.4. Analysis of Chlorin e6 and Rhodin g_7_ 7^1^-ethyl ester Uptake in MIA PaCa-2 and BV2 Cells

**Figure 6:**
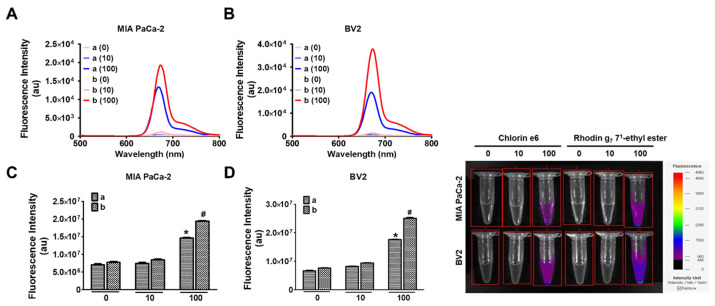
Cellular uptake of chlorin e6 or rhodin g_7_ 7^1^-ethyl ester observed using a plate reader and FOBI system in two different cell lines, MIA PaCa-2 and BV2 cells, after 3 h of post incubation. The cellular uptake of chlorin e6 and rhodin g_7_ 7^1^-ethyl ester (0, 10, and 100 μΜ) was measured using a plate reader (**A**,**B**) and FOBI system (**C**,**D**) in MIA PaCa-2 and BV2 cells, respectively. Data are presented as mean ± standard error of the mean *(n* = 3). * *p* < 0.05 compared to control for chlorin e6. # *p* < 0.05 compared to control for rhodin g_7_ 7^1^-ethyl ester (analyzed via one-way ANOVA with Tukey’s post hoc test). The concentrations of chlorin e6 (a) and rhodin g_7_ 7^1^-ethyl ester (b) were 0, 10, and 100 μM, respectively.

MIA PaCa-2 or BV2 cells in a 6-well plate were treated with chlorin e6 (**a**) or rhodin g_7_ 7^1^-ethyl ester (**b**) at 10 to 100 μΜ for 3 h, and their uptake was detected using a plate reader and the FOBI system. As shown in Figure 6A,B, the fluorescence of chlorin e6 or rhodin g_7_ 7^1^-ethyl ester-treated cell solutions increased with increasing concentration when evaluated using the plate reader in contrast to the untreated cells. The cells treated with a 100 μΜ dosage of chlorin e6 and rhodin g_7_ 7^1^-ethyl ester showed a significant increase in fluorescence intensity compared to that of the control. In BV2 cells, rhodin g_7_ 7^1^-ethyl ester exhibited a higher fluorescence intensity (2.2 and 2 times at 10 and 100 μΜ, respectively) than chlorin e6. In MIA PaCa-2 cells, rhodin g_7_ 7^1^-ethyl ester showed a higher fluorescence intensity (3 and 1.4 times at 10 and 100 μΜ, respectively) than chlorin e6. A similar pattern to a plate reader was found in the results measured by using the FOBI system (Figure 6C,D). Among the BV2 cells, rhodin g_7_ 7^1^-ethyl ester showed a higher fluorescence intensity (1.1 and 1.4 times at 10 and 100 μΜ, respectively) than chlorin e6. Similarly, in MIA PaCa-2 cells, rhodin g_7_ 7^1^-ethyl ester showed a higher fluorescence intensity (1.02 and 1.3 times at 10 and 100 μΜ, respectively) than chlorin e6. Therefore, the results of both the plate reader and FOBI showed that the cells treated with rhodin g_7_ 7^1^-ethyl ester had a higher fluorescence intensity than those treated with chlorin e6, indicating a greater uptake of rhodin g_7_ 7^1^-ethyl ester.

### 2.5. Analysis of Singlet Oxygen Photogeneration

**Figure 7:**
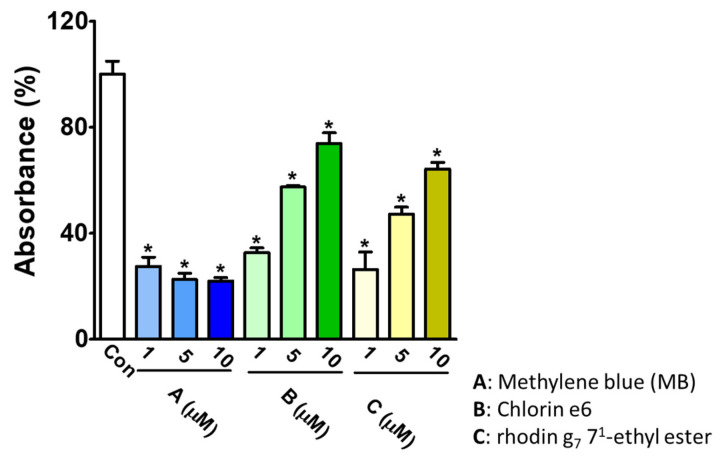
Evaluation of singlet oxygen mediated photooxidation of DPBF in the presence of methylene blue (A: MB), chlorin e6 (B), and rhodin g_7_ 7^1^-ethyl ester (C) at three different concentrations: 1, 5, and 10 µM. A light source emitting at 660 nm with a fluence rate of 5 mW/cm^2^ was utilized. Absorbance at 418 nm was measured and plotted against the concentrations of the various photosensitizers. The data presented are from a representative experiment with *n* = 3 replicates. * *p* < 0.05 compared to DPBF only (analyzed via one-way ANOVA with Tukey’s post hoc test for multiple comparisons).

The primary mechanism for the photodynamic death of cancer cells is singlet oxygen generation. Chlorin e6 (B) and rhodin g_7_ 7^1^-ethyl ester (C) were tested for their ability to produce singlet oxygen (^1^O_2_) under photosensitizing conditions in DMSO, using 1, 3-diphenylisobenzofuran (DPBF) as a specific ^1^O_2_ quencher. Methylene blue MB (A) was used as a positive control. The decrease in DPBF absorption intensity at 418 nm indicates the irreversible reaction between DPBF and ^1^O_2_ photogenerated by the tested compounds. When subjected to a 660 nm laser, a solution containing the most active rhodin g_7_ 7^1^-ethyl ester, chlorin e6, and DPBF suppressed the DPBF absorption band at 418 nm (Figure 7). At a concentration of 1 µM, MB produced the most significant ^1^O_2_ generation (22.22%) and generated constant values at all doses. Similarly, doses of 1 µM chlorin e6 (36.63%) and rhodin g_7_ 7^1^-ethyl ester (25.51%) showed the greatest decrease in DPBF absorbance indicating the highest increase in ^1^O_2_ generation. Interestingly, rhodin g_7_ 7^1^-ethyl ester generated 11.12% more singlet oxygen at a 1 µM concentration, compared to chlorin e6 alone. Chlorin e6 and rhodin g_7_ 7^1^-ethyl ester exhibited a large increase in DPBF absorbance at 5 and 10 µM dosages indicating a decrease in ^1^O_2_ generation.

### 2.6. Biodistribution Study

**Figure 8:**
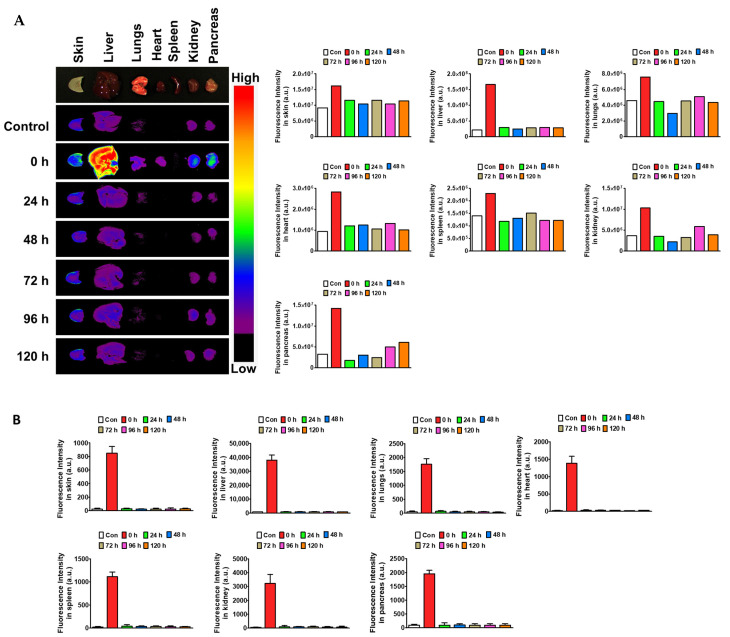
(**A**) Ex vivo organ analysis conducted in ICR mice at 0, 24, 48, 72, 96, and 120 h post intravenous administration of rhodin g_7_ 7^1^-ethyl ester at a dosage of 2.5 mg/kg, followed by quantitative analysis. The data are presented as the mean of a single experiment. (**B**) Biodistribution of rhodin g_7_ 7^1^-ethyl ester evaluated in lysates of resected organs at corresponding time points post injection. Quantitative analysis of fluorescence signals attributed to rhodin g_7_ 7^1^-ethyl ester is depicted in bar form. The data are presented as mean ± standard deviation (S.D.).

To determine the biodistribution of rhodin g_7_ 7^1^-ethyl ester, we measured ex-vivo FLI of excised organs in ICR mice after 0, 24, 48, 72, 96, and 120 h of intravenous drug treatment (Figure 8). After the respective time of incubation with 2.5 mg/kg of rhodin g_7_ 7^1^-ethyl ester, ex-vivo images were obtained. The figure demonstrates that the liver had a substantially greater rhodin g_7_ 7^1^-ethyl ester signal intensity than other organs, indicating its predominant accumulation in this highly vascular organ. Chlorin e6 also demonstrated similar indications in the liver [25]. The fluorescence assessments of rhodin g_7_ 7^1^-ethyl ester in a single organ revealed that it could concentrate substantially in numerous mouse tissues, such as that of chlorin e6. In addition, in all the organs, the fluorescence intensity peaked at 0 h post injection, while 24, 48, 72, 96, and 120 h of administration of rhodin g_7_ 7^1^-ethyl ester decreased the fluorescence due to the metabolic processes of the cellular system. However, in the case of chlorin e6, the fluorescence intensity was maximum at 0 h and greatly declined at 24 h, with a basal fluorescence level seen from 48 to 120 h post injection [25].

### 2.7. Detection of Reactive Oxygen Species (ROS)

**Figure 9:**
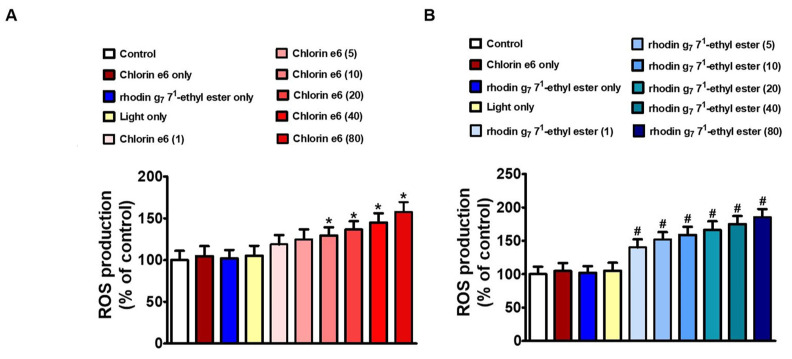
Comparison of ROS generation from chlorin e6-PDT and rhodin g_7_ 7^1^-ethyl ester-PDT at equivalent concentrations using DCFH-DA (*n* = 3). MIA-PaCa-2 cells were treated with varying concentrations (1–80 μΜ) of chlorin e6 or rhodin g_7_ 7^1^-ethyl ester for 3 h, followed by exposure to a 660 nm laser (power intensity of 0.5 J/cm^2^) for 1 min and 40 s. ROS production was quantified in (**A**) chlorin e6-PDT and (**B**) rhodin g_7_ 7^1^-ethyl ester-PDT, respectively. The data are presented as the mean ± SEM of triplicate samples. * *p* < 0.05 compared to the control for Ce6-treated groups. ^#^ *p*  <  0.05 compared to the control for rhodin g_7_ 7^1^-ethyl ester-treated groups.

In the following experiment, the determination of the ROS level related to chlorin e6-PDT or rhodin g_7_ 7^1^-ethyl ester-PDT in MIA-PaCa-2 pancreatic cancer cells was performed. The total ROS production was detected using DCFH-DA and is shown in Figure 9. The measured concentrations (1, 5, 10, 20, 40, and 80 μM) of both chlorin e6- and rhodin g_7_ 7^1^-ethyl ester-PDT revealed a dose-dependent increase in ROS. In both chlorin e6- and rhodin g_7_ 7^1^-ethyl ester-PDT, 80 μM produced the highest ROS, while 1 μM produced the lowest ROS. When compared among the two different photosensitizers, it was found that at all concentrations, rhodin g_7_ 7^1^-ethyl ester-PDT was likely to produce more ROS than chlorin e6-PDT. It is clear that rhodin g_7_ 7^1^-ethyl ester tends to produce higher ROS levels.

### 2.8. Cytotoxicity of Chlorin e6-PDT and Rhodin g_7_ 7^1^-ethyl Ester-PDT in Cancer Cells

The cytotoxicity in B16F10, MIA PaCa-2, PANC-1, HT-29, AsPC-1, and RAW264.7 cells was evaluated via an MTT assay 24 h following the separate administration of chlorin e6-PDT and rhodin g_7_ 7^1^-ethyl ester-PDT. As Table 1 indicates, the abovementioned cells were not inhibited by chlorin e6 or rhodin g_7_ 7^1^-ethyl ester alone or light alone. The inclusion of one normal cell line was used to test and compare the cytotoxic effects of chlorin e6 and rhodin g_7_ 7^1^-ethyl ester on normal and cancerous cells using their IC50 values. On the other hand, the cellular viability of one murine cancer cell line and four human cancer cell lines was examined using chlorin e6-PDT and rhodin g_7_ 7^1^-ethyl ester-PDT, and the results showed that the cell viability in relation to the photosensitizers varied. Both chlorin e6-PDT and rhodin g_7_ 7^1^-ethyl ester-PDT produced cytotoxicity in proportion to their concentration in all cell types. Figure 7 depicts the dark- and light-induced cytotoxic effects of chlorin e6 and rhodin g_7_ 7^1^-ethyl ester followed by illumination with a light dose of 0.5 J/cm^2^ in different cancers as well as normal cell lines. The established IC_50_ values are also summarized in Table 1. Post illumination, the IC_50_ values of chlorin e6-PDT ranged from 17.64 to 33.83 µM with the B16F10 cell line being the most sensitive and the PANC-1 cell line being the most aggressive. All cell lines showed ≥90% cell viability at a dose of 7.8 µM in contrast to the vehicle-treated control. For all cell lines, the cellular viability decreased with increasing doses and reached 10% at concentrations greater than 59.8 µM. We also determined the cytotoxic effect of photosensitizers in the complete absence of illumination, referred to as dark toxicity. The IC_50_ values changed to a greater concentration range of 250–564 µM with cellular viability ≥90% at concentrations as high as 169.2 µM. Further, the cellular viability decreased to less than 10% at concentrations up to 729.4 µM for all the cell lines. We also detected the photocytotoxicity and dark cytotoxicity of chlorin e6 in a normal RAW264.7 (macrophage) cell line, which showed less sensitivity with an IC50 of 45.4 and 455.9 µM, respectively.

**Figure 10:**
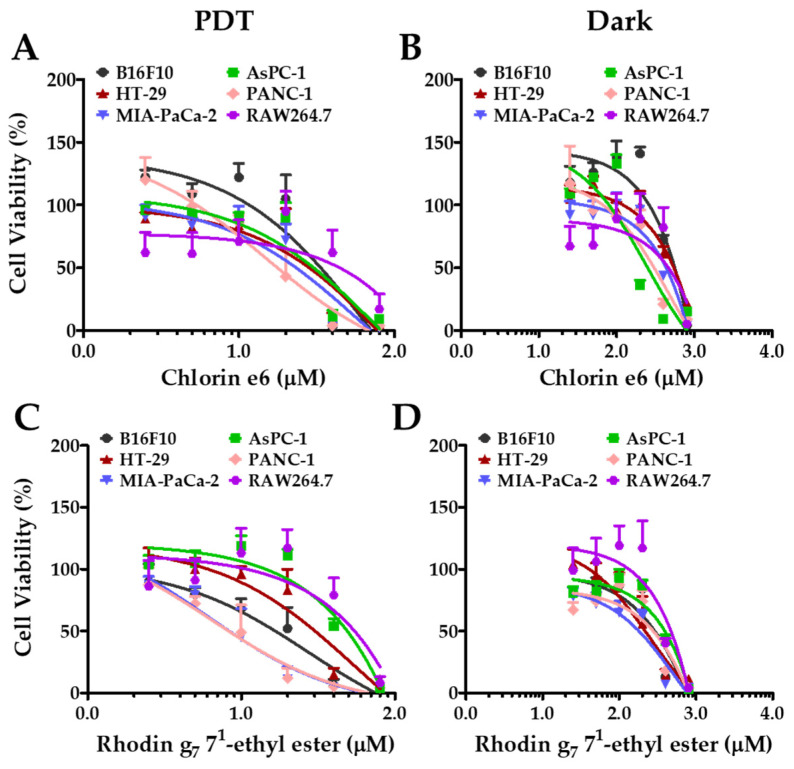
Evaluation conducted to assess the photo-induced and dark cytotoxicity in cancer cell lines treated with chlorin e6 and rhodin g_7_ 7^1^-ethyl ester. Panels (**A**,**C**) depict the photo-induced cytotoxicity profile of chlorin e6 and rhodin g_7_ 7^1^-ethyl ester in B16F10, MIA PaCa-2, PANC-1, HT-29, AsPC-1, and RAW264.7 cells, while panels (**B**,**D**) illustrate the dark cytotoxicity profile of chlorin e6 and rhodin g_7_ 7^1^-ethyl ester in the same cell lines.

Figure 10C illustrates the light-induced cytotoxic effects of rhodin g_7_ 7^1^-ethyl ester on five different cancer cell lines. The study was carried out by performing the MTT cell viability assay and via illumination with a light of 0.5 J/cm^2^ for 24 h. Following illumination, the AsPC-1 cell line was found to be the most sensitive, and the PANC-1 cell line was the most resilient with IC_50_ values ranging from 8.4 to 49.1 µM. When rhodin g_7_ 7^1^-ethyl ester was added at a concentration of ≥7.8 µM, all the cell lines showed ≥90% viability compared to the vehicle-treated control. With increasing concentrations of rhodin g_7_ 7^1^-ethyl ester, the cellular viability dropped to 10% at concentrations higher than 53.7 µM for all the cell lines. Most of the cancer cell lines were more sensitive to rhodin g_7_ 7^1^-ethyl ester-PDT than to chlorin e6-PDT. Similar to the dark cytotoxicity with chlorin e6, rhodin g_7_ 7^1^-ethyl ester’s IC50 values changed to a higher concentration range of 163–389 µM at concentrations as high as 37.4 µM, with the cellular viability of ≥90%. The cellular viability dropped to less than 10% at doses as high as 660 µM for all the cell lines. These findings showed that the phototoxic effect of rhodin g_7_ 7^1^-ethyl ester in combination with PDT is superior to that of chlorin e6-PDT. The photocytotoxicity and dark cytotoxicity of rhodin g_7_ 7^1^-ethyl ester in a normal RAW264.7 (macrophage) cell line also showed less sensitivity with an IC50 of 55.2 and 465.7 µM, respectively.

## 3. Discussion

The presence of an impurity in the active pharmaceutical ingredient poses challenges to the quality and safety of drugs. There is always a concern over the identification and its effect in biological cells and tissues. Consequently, the isolation and purification processes make them available in pure form for biological setups. In regard to impurity 4.5, reversed-phase medium pressure column chromatography enables us to obtain it in a very pure form as confirmed via HPLC. Since impurity 4.5 is slightly more polar than chlorin e6, our old samples of chlorin e6 contained high percentages of this impurity and were prepared from spirulina imported from China, which made purification and isolation easy. MPLC and HPLC are well-known tools to make purification and isolation easier. Our attempt to obtain a purified one by using a normal phase silica column was unsuccessful regarding the retention in the stationary phase (silica) lasting for a long time, leading to its degradation over time. The identical absorbance at the same excitation wavelength can be assumed to be absorbing similar photon energy. Thus, these candidate spectrums of impurity 4.5 can be utilized in photodynamic therapy. Similar absorbance and fluorescence, proton and carbon NMR, two-dimensional proton–proton correlation, and the overall verification of molecular weight via high-resolution mass spectrometry data of impurity 4.5 enable the comprehension of the structural determination, given the trivial name of rhodin g_7_ 7^1^-ethyl ester given to it. It is well known that rhodin g_7_ is a derivative arising from chlorophyll *b* [19,24]. However, this new process-related impurity is not directly formed from rhodin g_7_, but a series of reaction events take place at the aldehyde functional group of chlorophyll *b* as we proceed from extraction through pheophytin synthesis to the synthesis of chlorin e6. Further, rhodin g_7_ 7^1^-ethyl ester is a tricarboxylic acid having ethyl ester at C7 rather than at C13, C15, or C17. With regard to the real use of chlorophyll *b* and the related derivatives in PDT [26,27,28], rhodin g_7_ 7^1^-ethyl ester provides a new avenue for studies by medicinal chemists.

The synthesis of chlorin e6 involves solvent extraction and the alkaline hydrolysis of the ester group at C17^2^ considering that the release of the phytyl chain may increase the water-soluble ability. This sequence of reactions runs in parallel for chlorophyll *a* and chlorophyll *b*. Hence, the intermediates obtained from chlorophyll *b* via oxidation, pheophytinization, and alkaline hydrolysis were recorded for their HPLC-mass. It is noteworthy to mention that all the intermediates (**2**–**4**) leading to the formation of rhodin g_7_ 7^1^-ethyl ester were detected in the MS-ESI spectrum (Scheme 1). Rhodin g_7_ or the derivatives of chlorophyll *b* have rarely been used in photodynamic therapy [26,27,28]. While the esterifications of tricarboxylic acid groups (at C13, C15 and C17) in chlorin e6 via the reaction with alcohols in the presence of an acid are known, the esterification of rhodin g_7_ either at these carboxylic acids (C13, C15, and C17) or direct installing ethoxy (-OEt) at the carbonyl function group (-CHO) at C7 is unknown.

The ability to produce sufficient quantities of singlet oxygen is a critical feature of PDT sensitizers, which correlates with an increase in the lifespan of PS triplet excited states [29]. In addition, excessive amounts of ROS such as singlet oxygen, superoxide anion, and hydroxyl radicals induce damage to proteins, nucleic acids, lipids, and organelles, ultimately leading to the activation of regulated cell death pathways such as apoptosis in cancer cells [29,30]. We found that upon irradiation, rhodin g_7_ 7^1^-ethyl ester produced more singlet oxygen and intracellular ROS in comparison to chlorin e6. In addition, we also compared the cellular uptake of the two compounds. The cellular uptake and intracellular dispersion of drugs in cells rely on micro-environmental factors and cell features such as the capacity and affinity of the intracellular target sites to bind drugs and cells [31]. Our results from FOBI and the plate reader system suggest that at the maximum dose of 100 μΜ, the cellular absorption of rhodin g_7_ 7^1^-ethyl ester and chlorin e6 increased considerably. However, rhodin g_7_ 7^1^-ethyl ester demonstrated improved cellular absorption compared with chlorin e6 in both MIA PaCa-2 and BV2 cells. This rapid absorption of rhodin g_7_ 7^1^-ethyl ester implies that photoactive species are promptly delivered to the target locations, which is an important requisite for PDT. The precise position and controlled scope of the light irradiation are critical to achieving a targeted therapeutic effect against malignancies [28]. As a result, this intrinsic fluorescence of photosensitizers, acting as a bioprobe to inspect the vital tissue, was utilized to determine the time-dependent biodistribution of chlorin e6 and rhodin g_7_ 7^1^-ethyl ester. As shown by our prior observations, a high fluorescence signal of chlorin e6 was detected after 0 h of its intravenous injection, which diminished largely in 24 h and was reduced to the baseline at later time points [28]. In contrast, our current work found that the strong fluorescent signal of rhodin g_7_ 7^1^-ethyl ester was detectable only after 0 h post injection. Similar to chlorin e6, rhodin g_7_ 7^1^-ethyl ester was most abundant in the liver, followed by other organs.

We hypothesized that the cytotoxicity of rhodin g_7_ 7^1^-ethyl ester-PDT in different cancer cell lines would be enhanced in comparison to chlorin e6-PDT as the intracellular ROS in cancer cells was higher. In fact, we found that the IC50 values of rhodin g_7_ 7^1^-ethyl ester-PDT, in comparison to chlorin e6-PDT, were comparatively lower in relation to MIA PaCa-2, PANC-1, and B16F10 growth in vitro, demonstrating its superior phototoxic potential. However, rhodin g_7_ 7^1^-ethyl ester had no significant negative impacts on cell viability in the dark and was less phototoxic to normal cell lines. This suggests that rhodin g_7_ 7^1^-ethyl ester can efficiently produce cytotoxic species and cause laser irradiation-induced cell death. This higher phototoxic potency of rhodin g_7_ 7^1^-ethyl ester could be attributed to the greater singlet oxygen and intracellular ROS production, together with improved and faster cellular uptake. This suggests that adding functionality to chlorin e6 macrocycles maintains strong PDT efficacy. From the overall studies, we can infer that, as rhodin g_7_ 7^1^-ethyl ester exhibited promising PDT efficacy, it can be employed as a new photosensitizer for use in PDT. Research work on impurity 6.4 is currently underway in our laboratory at the Daegu cancer center and will be reported in due course.

## 4. Materials and Methods

### 4.1. General Information

Chlorin e6 samples were prepared from *Spirulina platensis* at Dongsung Cancer Center at Daegu, Republic of Korea. Commercially available starting materials and reagents were purchased from Sigma-Aldrich (Merck KGaA, Darmstadt, Germany), TCI Chemicals (Tokyo 103-0001, Japan), and Alfa-Aesar (Seoul, Suseo-dong, Suseo Officetel, Korea), and used without further purification. Biotech MPLC (Biotage Sweden AB, Box 8, 751 03 Uppsala, Sweden) as used for the purification of rhodin g_7_ 7^1^-ethyl ester. Thin-layer chromatography (TLC) was performed on silica gel plates (Kieselgel 60 F_254_, Merck) having a layer thickness of 0.25 mm. ^1^H NMR (600 MHz) and ^13^C NMR (125 MHz) spectra were recorded in Bruker using deuterated acetone. All the chemical shift values (*δ*) are expressed in parts per million (ppm) and coupling constants (*J*) in hertz (Hz). HRMS (Jeol_JMS-700, Tokyo 196-8558, Japan) was recorded at the Korea Basic Science Institute, Daegu.

HPLC analysis was performed on a Waters alliance 2695 utilizing a 2996 photodiode array detector (PDA) (Waters, Milford, MA, USA). HPLC separations were monitored at 407 nm. The following chromatography column was used: an analytical 150 mm × 4.6 mm column (Capcell Pak C18 UG120) packed with 5 µm particles. During the analysis, gradient elution was carried out using mobile phase A (0.1% TFA in water) and B (acetonitrile). Chlorin e6 and other impurities were separated using a 35-min gradient of B from 45 to 100%. A Waters Micromass (Milford, MA 01757, USA) ZQ single quadrupole mass spectrometer equipped with an ESI source was used for the LC-MS analysis.

### 4.2. Synthesis of Chlorin e6 and the Charracterization Data for Rhodin g_7_ 7^1^-ethyl Ester

Chlorin e6 was synthesized via the procedure mentioned in our earlier report. Reversed-phase column chromatography was performed multiple times using old samples of chlorin e6 containing a high content of impurity 4.5 after reversed-phase MPLC using a gradient solvent system. The desired compound was isolated at 36% of acetonitrile in 0.04% TFA in water. ^1^H NMR (600 MHz, acetone-*d*_6_) *δ* 9.93 (s,1H), 9.59 (s, 1H), 9.04 (s, 1H), 8.14 (dd, *J* = 18.0, 11.4 Hz, 1H), 6.37 (dd, *J* = 17.4, 1.2 Hz, 1H), 6.10 (dd, *J* = 11.4, 1.2 Hz, 1H), 5.59 (d, *J* = 19.2 Hz, 1H), 5.43 (d, *J* = 19.2 Hz, 1H), 4.66 (q, *J* = 7.2 Hz, 1H), 4.59 (d, *J* = 10.2 Hz, 1H), 4.44 (d, *J* = 15.0 Hz, 1H), 4.27 (d, *J* = 15.0 Hz, 1H), 3.80–3.70 (2H, m), 3.47 (s, 3H), 3.24 (s, 3H), 2.75–2.70 (m, 1H), 2.40–2.35 (m, 1H), 2.28–2.23 (m, 1H), 1.79 (d, *J* = 7.8 Hz, 3H), 1.67–1.63 (m, 7H); –1.17 (s, 1H, NH), –1.32 (s, 1H, NH); ^13^C NMR (150 MHz, acetone-*d*_6_) *δ* 174.7, 174.2, 171.6, 169.7, 169.2, 155.5, 149.4, 146.2, 140.3, 136.8, 136.7, 136.3, 135.4, 131.8, 130.4, 130.2, 122.3, 105.0, 98.8, 94.9, 72.7, 53.8, 50.0, 40.1, 39.0, 30.1, 30.0, 29.9, 29.7, 29.6, 23.3, 20.0, 18.0, 12.28, 11.26; HRMS (ESI): calculated for C_36_H_39_N_4_O_8_ [M+H]^+^ 655.2768; found 655.2771.

### 4.3. Analysis of Singlet Oxygen Photogeneration

Singlet oxygen photoregeneration was carried out using 1, 3-diphenylisobenzofuran (DPBF) in DMSO. DPBF is a selective ^1^O_2_ acceptor that reacts with ^1^O_2_. The sample solutions of DPBF (50 μM) which contained DPBF only (50 μM, as a control), MB (1, 5, and 10 μM), chlorin e6 (1, 5, and 10 μM), and rhodin g_7_ 7^1^-ethyl ester (1, 5, and 10 μM) were prepared under dark conditions. All of the samples were placed in a 96-well plate, which was covered with aluminum foil. The samples were then irradiated (660 nm, 50 mW, 0.5 J/cm^2^) for 1 min and 40 s, and after irradiation, the visible spectra of the sample solutions were measured spectrophotometrically. The normalized absorbance of DPBF at 418 nm was measured in these samples. The ^1^O_2_ photo-generation activities of MB, chlorin e6, and rhodin g_7_ 7^1^-ethyl ester can be compared with the different absorbance decay of each sample relative to the DPBF control sample.

### 4.4. Cell Culture

B16F10 (KCLB80008), MIA PaCa-2 (KCLB21420), PANC-1 (KCLB21469), HT-29 (KCLB30038), AsPC-1 (KCLB21682), and Raw 264.7 (KCLB40071) cells were procured from the Korean Cell Line Bank (KCLB, Seoul, Republic of Korea). AsPC-1 and RAW264.7 were thawed and maintained in RPMI-1640 media (Life Technologies corporation, Carlsbad, CA, USA) supplemented with 10% fetal bovine serum (Life Technologies corporation, USA) and 1% penicillin and streptomycin (Life Technologies corporation, USA), while MIA PaCa-2, PANC-1, HT-29, and B16F10 cell lines were cultured in DMEM (Life technologies corporation, USA) containing 10% fetal bovine serum and 1% penicillin and streptomycin. All the cells were cultured at 37 °C in a 5% CO_2_ incubator.

### 4.5. Detection of Reactive Oxygen Species (ROS)

MIA-PaCa-2 cells (density: 5 × 10^3^ cells/well) were seeded onto 96-well plates and incubated in a complete medium for 24 h at 37 °C. Then, the medium was replaced with a fresh culture medium containing chlorin e6 or rhodin g_7_ 7^1^-ethyl ester at different concentrations (1, 5, 10, 20, 40, and 80 μM) and then incubated for 3 h at 37 °C. After incubation, the cells were washed three times with PBS, and the cells were irradiated using a 630 nm laser at a power of 100 mW/cm^2^ for 300 s. After irradiation, a fresh culture medium containing 20 μM dichlorodihydrofluorescin diacetate (DCFH-DA) was added, and the cells were incubated for another 20 min. Fluorescence detection of DCFH-DA was performed via the fluorescence microplate reader using the Spark Multimode Microplate Reader (Tecan Trading AG, Männedorf, Switzerland).

### 4.6. Cellular Toxicity

After seeding B16F10 or MIA PaCa-2 at a density of 5 × 10^3^ cells/well and PANC-1, HT-29, or AsPC-1 at a density of 1 × 10^4^ cells/well in 96-well plates, the cells were incubated overnight at 37 °C in a CO_2_ incubator. The cells were exposed to different doses of either chlorin e6 or rhodin g_7_ 7^1^-ethyl ester (0, 2.5, 5, 10, 20, 40, and 80 µM) for 3 h. After the incubation, the cells were either subjected to a 660 nm laser with a power density of 50 mW (0.5 J/cm^2^) or not and were further incubated for 24 h at 37 °C. Moreover, the MTT assay was used to analyze the cell viability. The absorbance values of 540 nm were then measured using a microplate reader (Multiskan GO, Thermo Fisher Scientific, Oy, Ratastie 2, FI-01620 Vantaa, Finland).

### 4.7. Biodistribution Study

The animal study was approved by the Institutional Animal Care and Use Committee of the Dongsung Cancer Center under protocol IACUC # ds0022010107-2. The experiments were carried out in compliance with the ARRIVE guidelines. Male ICR mice (4 weeks old, OrientBio, Seongnam City, Republic of Korea) were used for the biodistribution study. The biodistribution analysis for rhodin g_7_ 7^1^-ethyl ester was calculated using tissue fluorescence. ICR mice (n = 7) were injected with or without rhodin g_7_ 7^1^-ethyl ester (2.5 mg/kg) intravenously. After the administration of rhodin g_7_ 7^1^-ethyl ester for 0, 24, 48, 72, 96, and 120 h, the mice were sacrificed, and their organs (kidney, spleen, heart, lungs, liver, skin, and pancreas) were resected. Fluorescence imaging was used to determine the ex vivo organ distributions of rhodin g_7_ 7^1^-ethyl ester utilizing the FOBI imaging system (Neo-Science, Suwon, Republic of Korea). For the rhodin g_7_ 7^1^-ethyl ester extraction, the organ tissues were then mixed with lysis buffer, homogenized in a tissue homogenizer, and centrifuged at 9000 rpm for 15 min. The rhodin g_7_ 7^1^-ethyl ester content in the supernatant was then evaluated using a Spark Multimode Microplate Reader (Tecan Trading AG, Männedorf, Switzerland) spectrophotometrically at 660 nm. The fluorescence intensity was adjusted to the tissue weight.

### 4.8. Analysis of Chlorin e6 and Rhodin g_7_ 7^1^-ethyl ester Uptake in MIA PaCa-2 and BV2 Cells

A quantitly of 5 × 10^5^ of MIA PaCa-2 and 2 × 10^5^ of BV2 cells per well were seeded in a 6-well plate and were treated with or without chlorin e6 or rhodin g_7_ 7^1^-ethyl ester (10 and 100 μΜ). After 3 h, the cells were harvested and washed twice with 1 × phosphate buffered saline (DPBS) to remove the remaining chlorin e6 or rhodin g_7_ 7^1^-ethyl ester from the culture media and cell surfaces. The cells from each well were resuspended in 1 mL of 1 × DPBS and measured using FOBI and a plate reader. chlorin e6 or rhodin g_7_ 7^1^-ethyl ester-treated cells were divided into triplicate wells of a black-walled 96-well plate, each containing 100 µL. The measurement was taken immediately after aliquoting using a Spark Multimode Microplate Reader (Tecan Trading AG, Männedorf, Switzerland) for spectroscopy. The fluorescence was measured using excitation and emission peaks of 405 nm and 500–800 nm. For the FOBI analysis, 700 µL of chlorin e6 or rhodin g_7_ 7^1^-ethyl ester-treated cells was added to the e-tubes, and bioimaging was performed immediately using the fluorescence-labeled organism bioimaging instrument (FOBI) system (Cellgentek, Daejeon, Republic of Korea). Each image was exposed for 0.3 s, with a 405 nm wavelength LED, and an emmission light of 502 nm. The results were further quantitatively measured using the “NEOimage FOBI” software.

### 4.9. Statistical Analysis

The obtained data are represented as mean ± standard deviation (SD) and were analyzed using one-way analysis of variance (ANOVA) with Tukey’s post hoc test, using GraphPad Prism software (version 5.01, Inc., 2007, San Diego, CA, USA). Probability values less than 0.05 indicate statistical significance.

## 5. Conclusions

In summary, a new process-related impurity, identified as rhodin g_7_ 7^1^-ethyl ester, was discovered during the synthesis of chlorin e6. This impurity, denoted as impurity 4.5, was characterized through HPLC/mass spectrometry and further confirmed via NMR and HRMS analysis. The methods for the purification and isolation of rhodin g_7_ 7^1^-ethyl ester have also been discussed. Based on the available HPLC/mass data, the potential pathway for the formation of rhodin g_7_ 7^1^-ethyl ester involving intermediates has been proposed. Studies have indicated a higher singlet oxygen generation, enhanced accumulation in highly vascular liver tissue, and increased production of reactive oxygen species in MIA-PaCa-2 cancer cells by using rhodin g_7_ 7^1^-ethyl ester. Cytotoxicity studies were conducted across five different cancer cell lines and a normal cell line for the first time, comparing the effects with those of chlorin e6. Rhodin g_7_ 7^1^-ethyl ester exhibited higher photo-induced toxicity and lower dark cytotoxicity in the tested cancer cell lines compared to chlorin e6. Notably, rhodin g_7_ 7^1^-ethyl ester demonstrated a nearly two-fold higher cytotoxicity in photodynamic therapy than chlorin e6. Consequently, rhodin g_7_ 7^1^-ethyl ester holds promise as a potential alternative photosensitizer or as a complementary agent to chlorin e6 in the photodynamic therapy for various diseases. However, preclinical research is needed to determine whether the rhodin g_7_ 71-ethyl ester-PDT has a similar anticancer effect in vivo.

## Figures and Tables

**Table 1:** Evaluation of PDT and dark cytotoxicity of chlorin e6 and rhodin g_7_ 7^1^-ethyl ester in five different cancer cell lines.

No.	Cell Line	Type	Chlorin e6 (µM)		Rhodin g_7_ 7^1^-ethyl Ester (µM)	
			PDT	Dark	PDT	Dark
1	B16F10	Murine melanoma	33.6 (±2.2)	555.7 (±6.1)	12.3 (±1.8)	262.0 (± 2.9)
		cancer cell				
2	HT29	Human colorectal cancer cell	28.1 (±1.4)	543.0 (±11.8)	30.1 (±3.8)	222.9 (±56.2)
3	MIA PaCa-2	Human pancreatic cancer cell	23.3 (±0.8)	430.3 (±4.7)	7.6 (±1.0)	160.7 (±1.8)
4	PANC-1	Human pancreatic cancer cell	17.9 (±1.8)	301.8 (±19.4)	8.4 (±1.6)	279.2 (±16.4)
5	AsPC-1	Human pancreatic cancer cell	30.4 (±2.1)	248.2 (±5.5)	49.1 (±0.9)	395.2 (±11.4)
6	RAW264.7	Murine Macrophage cell	45.4 (±9.0)	455.9 (±42.7)	55.2 (±3.4)	465.7 (±13.9)

## Data Availability

All the data are contained within the manuscript and Supplementary Materials.

## Supplementary Materials

The following supporting information can be downloaded at: https://www.mdpi.com/article/10.3390/ijms25137114/s1.
