# Supplementary material for: Isolation, Identification, and Biological Activities of a New Chlorin e6 Derivative
Source: Int J Mol Sci. 2024 Jun 28;25(13):7114. doi: 10.3390/ijms25137114 (PMC11240924; doi:10.3390/ijms25137114)
Supplement: Supplementary file 1 [file ijms-25-07114-s001.zip › ijms-3007080-supplementary.pdf]

Isolation, Identification, and Biological Activities of a New Chlorin e6 Derivative

Rameshwar Prasad Pandit [1], Til Bahadur Thapa Magar [2], Rajeev Shrestha [3], Junmo Lim [1], Pallavi Gurung [1] and Yong-Wan Kim [1,*]

[1] Dongsung Cancer Center, Dongsung Biopharmaceutical, Daegu 41061, Republic of Korea; prp08@ds-pharm.co.kr (R.P.P.); ijm15@ds-pharm.co.kr (J.L.); gp20@ds-pharm.co.kr (P.G.)
[2] Center for Translational Science, Florida International University, 11350 SW Village Pkway, Port St. Lucie, FL 34987, USA; laphale@outlook.com
[3] Center for Food Animal Health, Department of Animal Sciences, The Ohio State University, Wooster, OH 44691, USA; shrestha.144@osu.edu
* Correspondence: thomas06@hanmail.net

Contents

HPLC of chlorin e6 (purchased from Frontier): S1

Structural elucidation by ca*rbon NMR of impurity 4*.5: S2

A*nalysis of chlorin e6 and rhodin g_7 7[1]-ethyl ester uptake in MIA PaCa-2 and BV2 cells* (Cellular uptake of chlorin e6 and rhodin g_7 7[1]-ethyl ester): S3

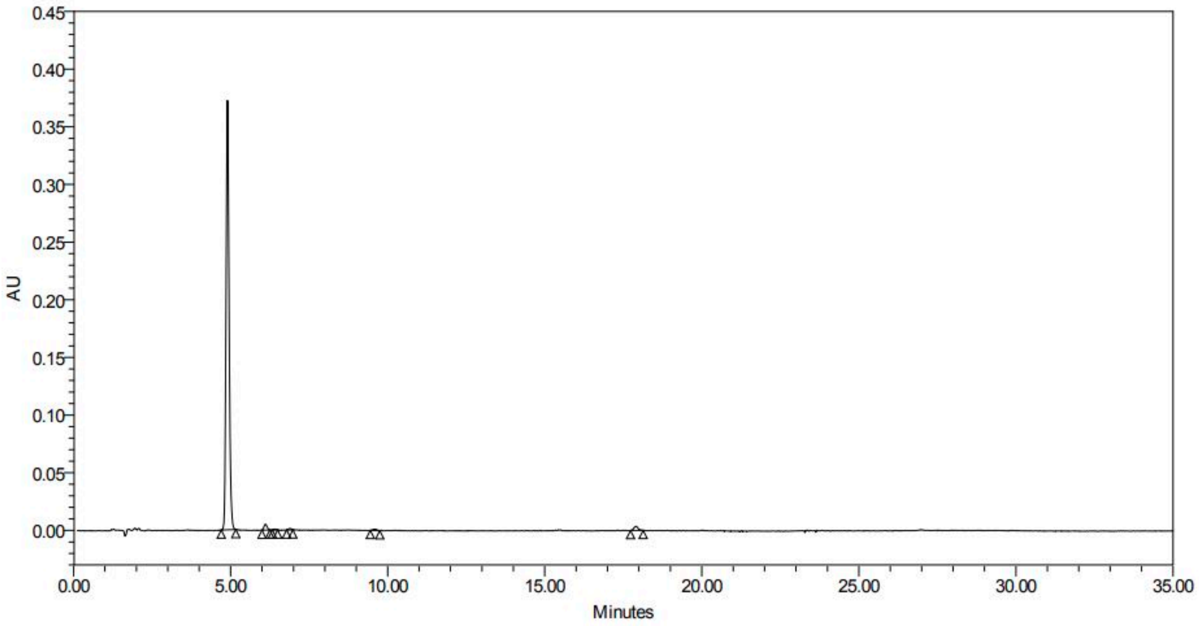

	RT	Area	% Area	Height
1	4.901	2479014	96.41	369648
2	6.111	35691	1.39	5092
3	6.395	4664	0.18	812
4	6.884	7871	0.31	1189
5	9.595	10473	0.41	1005
6	17.910	33656	1.31	3568

Figure S1. HPLC of chlorin e6 (purchased from Frontier). HPLC condition were same to that of mentioned in manuscript.

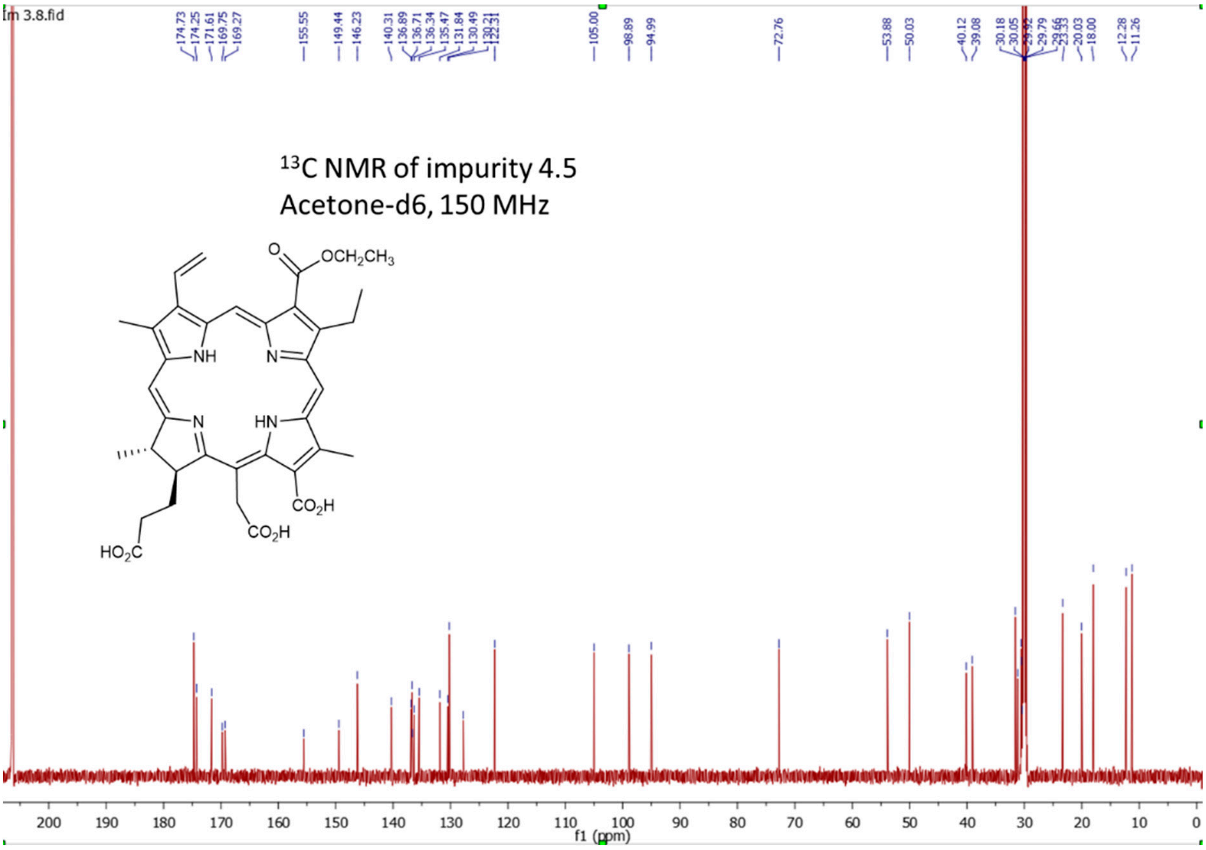

Figure S2. ^{13}C NMR of impurity 4.5.

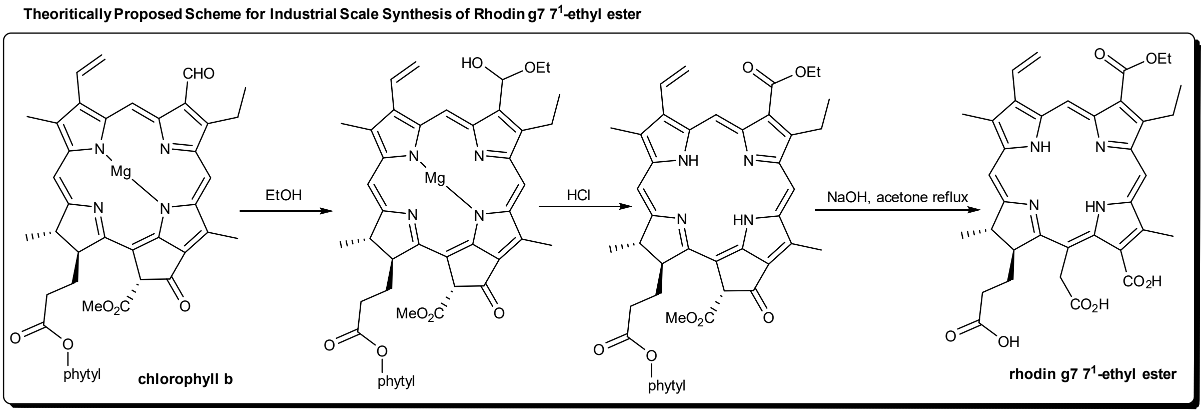

Figure S3. Theoritically proposed scheme for industrial synthesis.
